# Gilles de la Tourette Syndrome: advice in the times of COVID-19

**DOI:** 10.12688/f1000research.23275.2

**Published:** 2020-04-28

**Authors:** Mary M. Robertson, Valsamma Eapen, Renata Rizzo, Jeremy S. Stern, Andreas Hartmann

**Affiliations:** 1 Department of Psychiatry, University College of London, London, W1T-7NF, UK; 2 University of New South Wales and Academic Unit of Child Psychiatry, Liverpool Hospital and Ingham Institute, Sydney, NSW 2170, Australia; 3 Section of Child and Adolescent Neuropsychiatry, University of Catania, Catania, Italy; 4 Department of Neurology, St George’s University of London, London, SW17 0QQ, UK; 5 Department of Neurology, Groupe Hospitalier Pitié-Salpêtrière, Paris, 75013, France

**Keywords:** Gilles de la Tourette syndrome, COVID-19, tics, anxiety, OCD, ADHD, confinement

## Abstract

The novel coronavirus disease (COVID-19) was identified as the cause of an outbreak of respiratory disease in China at the end of 2019. It then spread with enormous rapidity and by mid-March 2020 was declared a world pandemic. Gilles de la Tourette Syndrome (GTS) is a childhood-onset neurodevelopmental disorder with a worldwide prevalence of about 1% of the population. The clinical symptoms include multiple motor and one or more phonic (vocal) tics. Germane to this communication is that 85% of patients with GTS have associated psychiatric co-morbidities, many of which are being exacerbated in the current global health crisis. In addition, several symptoms of GTS may mimic COVID-19, such as a dry cough and sniffing (phonic tics), while other symptoms such as spitting, inappropriate touching of others and “non-obscene socially inappropriate symptoms” can potentially get patients with GTS into trouble with the law. We suggest that a clear explanation of the COVID-19 illness and GTS is important to enable colleagues of various specialities who tend to patients with GTS. It is important to acknowledge at the outset that the information available on the COVID-19 pandemic changes daily, including cases infected, deaths reported, and how various national health systems are planning and or coping or not. It is fair to say that having read the current medical and lay press we conclude that it is not easy to reassure our patients with absolute certainty. However, notwithstanding that, we hope our documentation is of some assistance.

## Aetiology: COVID-19

Coronaviruses are a group of viruses that mostly result in mild illnesses, similar to the “common cold”, targeting the upper respiratory tract. However, certain types of coronaviruses can affect the lower airway, causing serious illnesses such as bronchitis or pneumonia. Coronaviruses have very large single stranded RNA genomes (approximately 26-32 KB). Coronavirus particles are surrounded by a fatty outer layer called an “envelope” and usually are spherical when visualised under an electron microscope, with a “corona” of club-shaped spikes on their surface ( Wu *et al.*, 2020). Most people become infected with coronaviruses at some point in their lives, but the majority of these are harmless. The novel coronavirus, which causes the COVID-19 illness, is different, and can be extremely serious.

The virus that causes COVID-19 is known as SARS-CoV-2 (Severe Acute Respiratory Syndrome Corona Virus 2). It appears to have emerged in Wuhan, Hubei Province, China. The viruses first inhabited animals such as bats and then “jumped” to humans: this occurred in China in late 2019, from whence it has spread worldwide ( Zhu *et al.*, 2020). Coronaviruses replicate their RNA genomes using enzymes called RNA-dependent RNA polymerases, which are prone to errors, but genomic analysis to date suggests that SARS-CoV-2 is mutating slowly, thus reducing the chance of it changing to become deadlier ( Wu *et al.*, 2020). It is worth mentioning that since 2003, viral diseases have caused major pandemics. These include the coronaviruses, which have caused multiple major public health events that resulted in global pandemics such as severe acute respiratory syndrome (SARS; or “bat SARS”), Middle East respiratory syndrome (MERS) and the current coronavirus disease (COVID-19) ( Kandeel *et al.*, 2020). In addition, with a growing threat to maternal-foetal health, the Zika viral illness began in 2009 with devastating consequences and continues to spread ( Towers *et al.*, 2018). Finally, in some parts of the globe, cases of swine flu (H1N1 virus) are still on the rise such as in India, where in 2020 alone around 1500 cases have been reported to date, with 28 deaths in the period from 1 January to 1 March 2020.

## Clinical features: COVID-19

COVID-19 can affect individuals of any ages and clinical symptoms may vary. The main recognised clinical features of COVID-19 include fever (52% of younger patient, 89% of older patients), cough (44% younger, 77% older; usually dry, but can be expectorant), fatigue or tiredness; more severe cases get pneumonia. In mild cases people may have only a runny nose or sore throat. Patients may also have gastro-intestinal symptoms such as nausea, diarrhoea and anorexia. It does appear that older people, on the whole, are more severely affected. Individuals with pre-existing co-morbid conditions also appear to be particularly at risk: these illnesses include hypertension, diabetes, cardiovascular disease, and malignancy ( Mao *et al.*, 2020; Wan *et al.*, 2020; Zhang *et al.*, 2020a; Zhang *et al.*, 2020b; Zhang *et al*., 2020c; Zheng *et al.,* 2020a). There is also recent evidence that anosmia or loss of smell is a symptom of COVID-19 and can even be the presenting symptom ( Hopkins & Kumar, 2020; Lüers *et al.*, 2020). Recent unpublished results in a series of 417 patients suggest rates of >80%, and a large European multicentre trial is currently being undertaken.

Severity has been described in various ways. Patients with abnormal chest images are older, with a higher rate of pre-existing conditions, lower rate of exposure history, and a longer time between onset and confirmation than patients without pneumonia. In addition, patients with more severe symptoms have a higher rate of fever, expectorant cough and headache, lower lymphocytes, albumin and serum sodium, and a higher total bilirubin, c-reactive protein, creatine kinase and lactate dehydrogenase, ( Zhang *et al.*, 2020c). Muscle aches, headache, shortness of breath and nausea/vomiting are also associated with disease severity. Also, biomarkers such as lower lymphocyte levels in addition to raised leukocytes, D-dimer levels, procalcitonin and serum creatinine, as well as higher radiograph score at admission are predictive factors for a severe/critical subtype ( Zhang *et al.*, 2020c). Finally, it has been shown that elevated exhaustion levels and reduced functional diversity of T cells in peripheral blood may predict progression in COVID-19 patients ( Zheng *et al*. 2020b). In the most severe cases, patients go into respiratory and multi-organ failure. It must be borne in mind, however, that mortality has also been reported in children, adolescents and young adults. Thus, it does appear that no one is immune or not at risk.

## Aetiology and epidemiology: Gilles de la Tourette Syndrome

Gilles de la Tourette syndrome (GTS) is a complex neurodevelopmental disorder that has aetiological contributions from genetic influences in pregnancy and birth difficulties, and immunological variables ( Robertson *et al.*, 2017). Whereas the PANDAS (Pediatric Autoimmune Neuropsychiatric Disorders Associated with Streptococcal Infections) hypothesis is increasingly being discarded, an immunological trigger is possible in a subset of patients with GTS as they might show differences in immune responses compared to controls ( Martino *et al.*, 2015); however, how these might possibly affect response to viral infections and, more specifically SARS-CoV-2, remains as yet unknown (see below).

## Clinical features: Gilles de la Tourette syndrome

The generally recognised diagnostic criteria according to the DSM-5 for GTS are multiple motor and at least one or more vocal (phonic) tics over the course of at least 12 months in a patient less than 18 years old ( APA, 2013). The phenotypic spectrum also includes chronic (persistent) motor or vocal tic disorder and transient (provisional) tic disorder. The most common motor tics are facial (such as eye blinking and grimacing), whereas the most common phonic tics are coughing, sniffing and throat clearing ( Robertson *et al.*, 2017). Individuals may have the relatively rare (around 15–20%) but “press-worthy” symptom of coprolalia ( Freeman *et al.*, 2009).

Neuropsychiatric comorbidities are common (around 85% of patients) and include obsessive-compulsive disorder or behaviours (OCD/OCB), attention-deficit hyperactivity disorder (ADHD), learning disabilities, depression, anxiety, and autism ( Rizzo *et al.*, 2014; Robertson *et al.*, 2017).

It is also worth noting that quality of life in patients with GTS has been demonstrated to be reduced ( Elstner *et al.*, 2001; Eddy *et al.*, 2011). In addition, Eapen *et al.* (2016) reviewed the literature, pointing out that both tics and comorbidities in GTS may result in poorer psychosocial functioning. Much of the stigma and social maladjustment associated with GTS resulting in exclusion, bullying and discrimination, can be largely caused by misperceptions of the disorder by teachers, peers and the wider community. Thus, in the current “COVID-19 climate,” we must be mindful to remain attuned to the needs of our patients with GTS.

## Gilles de la Tourette syndrome in the context of COVID-19

Potential consequences of the COVID-19 pandemic on patients with GTS can be classified according to four axes:

1. Anxiety related to the pandemic
2. Confinement/quarantine
3. Alterations in tics and behaviours specific to GTS
4. Neurotropic effects of SARS-CoV-2

### Anxiety related to the pandemic

Anxiety is a frequent comorbidity in GTS, manifesting both as generalized anxiety as well as specific phobias ( Martino *et al.*, 2017). Obviously, the relentless and generally starkly pessimistic media coverage on COVID-19 is likely to exacerbate these anxieties. In young children, their comprehension of the situation is limited, but the transmission of parental anxiety is a possible concern, either because of the pandemic itself and/or the economic consequences they face.

OCD is an equally frequent comorbidity in GTS. In the current era of governmental and public health instructions designed to slow the spread of COVID-19, patients with contamination OCD are at particular risk of experiencing conflicting demands since their baseline cognitive-behavioural training (CBT) (if applicable) asks for less hand washing, the exact opposite of what is now being recommended: this may result in cognitive dissonance which may turn close to unbearable. Obsessions relating to death (one’s own or loved ones) will likely also rise, as well as morbid ruminations ( Robertson & Cavanna, 2007), and possibly phenomena typical of GTS such as symmetry and ‘just right’ behaviours.

In addition, anxiety may trigger an increase in tic severity. It must be explained to patients and parents that this does not reflect an aggravation of GTS *per se* but is contextual, and thus likely reversible in the foreseeable future. An exacerbation of baseline tics in general may not be inevitable. In normal practice patients sometimes even report a *reduction* of GTS symptoms in association with major anxiety-provoking life events, although the reverse is conventionally more often true. Aside from OCD, entire populations are now undergoing some kind of “life event”, no doubt to be the subject of major study in due course.

### Confinement/quarantine

The psychological sequelae of confinement and quarantine following the COVID-19 outbreak have been well reviewed recently ( Brooks *et al.*, 2020). The authors reviewed the psychological impact of quarantine using three data bases and 3166 papers out of which 24 were included. Most studies reviewed reported negative psychological effects including post-traumatic stress symptoms, confusion and anger. Stressors included longer quarantine duration, infection fears, frustration, boredom, inadequate supplies, inadequate information, financial loss and stigma. Some researchers suggested a long-lasting impact.

As with anxiety – since confinement and quarantine are anxiety-triggering in their own right – an increase in tic severity can be expected. Moreover, behavioural abnormalities will likely also be exacerbated. First comes ADHD, present in 50–70% of patients with GTS ( Hirschtritt *et al.*, 2015). In particular, the hyperactive phenotype makes confinement and quarantine hard to bear as physical energy cannot be released. It is also likely that rage attacks/explosive outbursts, as well as oppositional behaviour – which largely depend on the presence of comorbid ADHD ( Müller-Vahl *et al.*, 2020) – will put an increasing strain on families. Reducing screen time – a frequent trigger of explosive outbursts – follows a paradoxical injunction similar to hand washing since it is more difficult to enforce in conditions of confinement and quarantine, added to which comes reduced schooling time. Yet, this is a potential conflict for parents as allowing children and adolescents to use screens more abundantly than usual may be the only way to keep the peace for the time being. Nevertheless, even in instances where increased screen time cannot be avoided, it is still possible to ensure that the content of what is being watched is age appropriate, engaging/interactive and meaningful. With regard to ADHD and impulsivity, following the rules (hygiene and social distancing) may be particularly difficult. Note that behavioural abnormalities related to ADHD not only concern children but also adults, especially maladaptive behaviours ( Haddad *et al.*, 2009). Home schooling may further penalize children with learning disabilities, frequent in GTS. Lastly, a hallmark of GTS is individual variation. We have also heard from adult patients whose tics are usually worse when they are out in society and less intrusive at home; some have described being in self-isolation with families or alone and finding their tics relatively settled.

### Alterations in tics and behaviours specific to GTS

As outlined in the two previous sections, many problems that patients with GTS face in the context of COVID-19 are related to their comorbidities, with an increase in tics the only outcome specific to their underlying disorder. However, certain nosographic features unique to GTS must also be considered.

Firstly, non-obscene socially inappropriate symptoms (NOSIS) ( Eddy & Cavanna, 2013; Kurlan *et al.*, 1996) which lead patients to display behaviours at odds with what is socially acceptable and can also be referred to as provocative and transgressive. Patients told us of developing new coughing tics, with a particular urge in public. Other examples to be expected in times of COVID-19 may manifest as spitting, sneezing into one’s hands, not maintaining social distancing, or attempting to shake hands (personal observations). Touching other people is not an uncommon complex motor tic but in the “COVID-19 climate” could also represent NOSIS (see Table 1). Note that these behaviours can also fall into the risk-seeking/impulsive phenotype often present in patients with GTS ( Wright *et al.*, 2012). Furthermore, echolalia and echopraxia may lead patients with GTS to copy other people’s coughing. In case of palilalia and palipraxia (repeating their last actions), a first ‘real’ cough may be repeated over and over again without any underlying respiratory need.

**Table 1.** Summary of the demography and clinical features of GTS and COVID-19.

Clinical issues	Tourette syndrome	COVID-19
History	Documented in the medical literature since 1800s and the symptomatology has remained the same	First described in China in December 2019
Aetiology	Complex - Genetic - Birth difficulties - Immune factors	Viral infection – SARS-CoV-2
Infectious	Not at all	Highly
Epidemiology	Worldwide	Worldwide
Fatality	Not *per se* (but suicide rate is higher than in the general population)	High (2–4%)
Age at onset	Young (peak 5–8 years)	Any ages affected
Age difference in severity of symptoms	Young people – mild on the whole Severity reduces with age – even in an individual	Young and fit are usually mild Worst severity in - Older people - People with cardiovascular/metabolic risk factors) - Immune deficiencies
Clinical symptoms (either mistaken for or pose difficulties in a Covid-19 world)	Vocal tics - Coughing - Sniffing - Throat clearing Motor tics - Eye blinking - Head nodding Echopraxia/Palilalia e.g. copying others (e.g. coughing) Palilalia/Palipraxia – repeating action or sound (e.g. coughing) Forced touching of others	- Dry cough - High Fever - Difficulty breathing
Clinical severity and impairment Worse with comorbidity and co-existent disorders and others features	Comorbid - OCD/OCB - Autism - ADHD Co-existent - Anxiety - Depression - Phobias Other features important - NOSI - SIB	- Worst severity in those with comorbid conditions, especially 1. respiratory 2. cardio-vascular 3. reduced immunity
Management and treatment	Complex with - Medication - CBT - DBS (rare)	- Nil specific - Many agents tried - Severely ill patients require ICU and ventilators - Some assisted heart & lung

### Neurotropic effects of SARS-CoV-2

To date, little is known about the potential neurological effects of SARS-CoV-2 but these have been suggested ( Baig *et al.*, 2020) and there is an evolving observation of high rates of anosmia and hypogeusia even in mild infection which may indicate neurotropism ( Hopkins & Kumar, 2020; Lüers *et al.*, 2020). Undoubtedly, this literature will grow over the upcoming months and years. As immune function may be altered (even though this does not necessarily mean diminished or compromised) in GTS, one may wonder whether our patients bear a special risk of sequelae. Intuition however dictates that this is highly unlikely as viral CNS infections have a higher probability to target people with neurodegenerative disease, or even causing it, as was the case after the Spanish influenza pandemic and resulting Encephalitis Lethargica ( Hoffman & Vilensky, 2017). In neurodevelopmental disorders such as GTS – and this also holds true for its comorbidities which are based on circuit dysfunctions – it is, at present, hard to see how a viral infection, and more specifically SARS-CoV-2, could lead to lasting CNS sequelae specific to GTS (for an evolving online resource, see here). However, only time will tell.

## Management and treatment

Accurate recognition of the symptoms of coughing or sniffing as related to GTS as opposed to COVID-19 is critical in providing the right intervention and support. Similarly, spitting or forced touching etc. can be part of tic-related behaviours that may get the patient in trouble with the law. Hence awareness and appropriate support (e.g. the GTS patient may have to carry a card, informing authorities and other appropriate people about the condition) is important. Further, accurate identification and treatment of tics and comorbidities should continue as usual and according to established guidelines, if possible ( Pringsheim *et al.*, 2019). It is likely that medications will need to be increased probably transiently until the COVID-19 crisis resolves. That is obviously true for anti-tic medication as it is for psychostimulants (for ADHD) and selective serotonin reuptake inhibitors (for anxiety and OCD). There are currently unprecedented challenges in healthcare delivery and some of the specialists who would normally give this advice in many countries may be unavailable for a time due to redeployment, potentially causing further stress to families. Also, CBT for tics ( van de Griendt *et al.*, 2013) should be maintained by videoconferencing whenever possible ( Andrén *et al.*, 2019). The same applies to supportive psychotherapy for those who suffer from anxiety, depression, and loneliness. Finally, telehealth will play an increasing role in the medical follow-up of patients with TS, likely beyond the end of the pandemic. It will be important to establish whether this type of care will be well accepted by patients and families alike.

The National Patient Advocacy Groups for GTS in the UK and USA have published factual information and advice for individuals with GTS, their families and health-care professionals. These represent invaluable resources in their own right and, moreover, underline a sense of (global) community that is vital in these times.

Concerning the points raised above, we propose the following measures:

- Reduce media coverage to curb anxiety.
- Limit parental anxiety (or the expression thereof) as much as possible.
- In patients with contamination OCD, relax CBT rules in collaboration with the therapist. Insist that this is a temporary measure.
- Explain, if necessary, that tic increase does not reflect an aggravation of the primary disorder but is contextual, and thus transient.
- Reduce screen time reasonably (i.e. without triggering rage attacks and to avoid excessive boredom), but more importantly focus on the content of what is being watched.
- Use outdoor activities as much as possible and legally acceptable, especially when ADHD is present, or engage in activities that can be done indoors.
- Insist on upholding current rules (hygiene, social distancing) firmly but also gently.
- If patients get in conflict with the law or just other individuals because of NOSIS, echo- or palipraxia (i.e. abundant coughing in public or displaying at-risk behaviours), attempts should be made to resolve the issue by explaining these symptoms. In some cases, “urgency cards” can also be of great help (see here and here).
- For health-care professionals: keep up outpatient clinics by telephone or videoconferencing as much as possible. The latter may prove useful even after the pandemic will have abated.
- Finally, there are no indications that SARS-CoV-2 infections might lead to central nervous system sequelae for patients with GTS in the intermediate or long term. Neither does GTS alone put individuals in a higher risk group for general consequences of infection. If necessary, reassurance is needed in this matter.

## Conclusion

GTS, as a complex neuropsychiatric disorder, offers many angles of attack for the current COVID-19 pandemic and its consequences (social distancing, home schooling, confinement/quarantine, and living in a general climate of fear). Also, some patients might expose themselves to social conflict because of behavioural features specific to GTS. Thus, they need our reassurance and help in these difficult times, supported by a network of informed health-care professionals, although already rare in ‘normal’ times. Ideally, specialist medical services will be able to organise in the face of acutely changing roles in hospitals to provide advice when needed, to avoid a feeling of professional abandonment (which obviously is not special to patients with GTS). With this short paper, we hope to have contributed a few small pieces to the puzzle which will hopefully help us navigate through the upcoming weeks and months.

## Data availability

The data referenced by this article are under copyright with the following copyright statement: Copyright: ï¿½ 2020 Robertson MM et al.

Data associated with the article are available under the terms of the Creative Commons Zero "No rights reserved" data waiver (CC0 1.0 Public domain dedication). 

No data are associated with this article.
