# Supplementary material for: Gilles de la Tourette Syndrome: advice in the times of COVID-19
Source: F1000Res. 2020 Apr 28;9:257. Originally published 2020 Apr 14. [Version 2] doi: 10.12688/f1000research.23275.2 (PMC7195896; doi:10.12688/f1000research.23275.2)
Supplement: Peer review history

# Peer review: Reviewer response for version 2

**DOI:** 10.5256/f1000research.26007.r62824

**Published:** 2020-04-28

**Authors:** Andrea Cavanna

**Affiliations:** 1 Department of Neuropsychiatry, BSMHFT, University of Birmingham, Birmingham, UK

Approved.

Is the topic of the opinion article discussed accurately in the context of the current literature?

Yes

Are arguments sufficiently supported by evidence from the published literature?

Yes

Are all factual statements correct and adequately supported by citations?

Yes

Are the conclusions drawn balanced and justified on the basis of the presented arguments?

Yes

Reviewer Expertise:

Behavioural neurology and neuropsychiatry.

I confirm that I have read this submission and believe that I have an appropriate level of expertise to confirm that it is of an acceptable scientific standard.

# Peer review: Reviewer response for version 1

**DOI:** 10.5256/f1000research.25696.r62277

**Published:** 2020-04-17

**Authors:** Andrea Cavanna

**Affiliations:** 1 Department of Neuropsychiatry, BSMHFT, University of Birmingham, Birmingham, UK

This is a timely, informative, and well written article providing clinicians with practical advice about how to best advise and support patients with tics during these challenging times. The authors navigate through the many uncertainties of the unfolding circumstances using the best available evidence as well as their own invaluable experience, according to the ancient motto ‘scientia et conscientia’.

 I have little to say in terms of further improving this useful article. As the authors rightly point out, anxiety is a common comorbidity that needs to be addressed with particular care, in consideration of its recognized role as tic-exacerbating factor.

 A recent review article on telemedicine for hyperkinetic movement disorders covers tic disorders, and states that “the extent to which telemedicine facilitates multidisciplinary treatment needs to be further studied” (Srinivasan et al. 2020) ^1^. It is hoped that the current circumstances and the unprecedented measures (including social distancing) are also seen as an opportunity to improve our understanding of patients’ needs. The authors might want to add their suggestions about gathering high quality information on remotely delivered clinical input.

Is the topic of the opinion article discussed accurately in the context of the current literature?

Yes

Are arguments sufficiently supported by evidence from the published literature?

Yes

Are all factual statements correct and adequately supported by citations?

Yes

Are the conclusions drawn balanced and justified on the basis of the presented arguments?

Yes

Reviewer Expertise:

Behavioural neurology and neuropsychiatry.

I confirm that I have read this submission and believe that I have an appropriate level of expertise to confirm that it is of an acceptable scientific standard.

## References

[1] : Telemedicine for Hyperkinetic Movement Disorders. *Tremor Other Hyperkinet Mov (N Y)*.2020;10: 10.7916/tohm.v0.698 10.7916/tohm.v0.698 32195039PMC7070700

# Peer review: Reviewer response for version 1

**DOI:** 10.5256/f1000research.25696.r62276

**Published:** 2020-04-16

**Authors:** Kirsten R Müller-Vahl

**Affiliations:** 1 Clinic of Psychiatry, Social Psychiatry and Psychotherapy, Hannover Medical School, Hannover, Germany

This is a nicely written paper by a group of renowned European Tourette experts about GTS in times of COVID-19. The authors discuss similarities of COVID-19 and tics in GTS and outline specific problems that may result from the pandemic for this group of patients. Finally, they give concrete suggestions how to advice and treat patients with GTS and their families during the pandemic.

 I like this small paper and find it interesting to read, since it alerts us that the current pandemic may be much more challenging for patients with GTS compared to healthy people.

 I have some minor suggestions the authors may take into consideration to further improve the quality of this manuscript:

- The authors mix the name of the virus (SARS-CoV-2) and the name of the disease caused by this virus (COVID-19). COVID-19 is simply the abbreviation of Coronavirus *Disease* 2019. Therefore, the authors should correct for example the heading „ Aetiology: COVID-19 (coronavirus)”. As far as I know, the correct spelling is “SARS-CoV-2” (not “SARS-Cov-2”). Also in Table 1: “Viral infection – Coronavirus 19“ please change in “SARS-CoV-2”.
- The authors describe the different symptoms associated with COVID-19 including neurological complications. I suggest to include recent reports on meningitis/encephalitis (for example see: https://doi.org/10.1016/j.ijid.2020.03.062) ^1^.
- Coprolalia: according to the literature it occurs in more than 10% of patients. I suggest to include a reference here too.
- The authors should use the term “pandemic” throughout instead of in some cases “epidemic”.
- Correct “cognitive-behavioural (CBT) training” in “cognitive-behavioural training (CBT)”.
- Rephrase this sentence (I think the “a” must be deleted): “Touching other people is a not an uncommon complex motor tic but in the “COVID-19 climate” could also represent NOSIS (see Table 1).”
- Table 1: I suggest to spell “Palilalia/Palipraxia” and “Echo praxia – lalia” in the same way.
- I suggest to change the order: “selective serotonin reuptake inhibitors (SSRIs)” instead of “SSRIs (selective serotonin reuptake inhibitors)”.

Is the topic of the opinion article discussed accurately in the context of the current literature?

Yes

Are arguments sufficiently supported by evidence from the published literature?

Yes

Are all factual statements correct and adequately supported by citations?

Yes

Are the conclusions drawn balanced and justified on the basis of the presented arguments?

Yes

Reviewer Expertise:

Tourette syndrome, tic disorders, cannabis-based medicine

I confirm that I have read this submission and believe that I have an appropriate level of expertise to confirm that it is of an acceptable scientific standard.

## References

[1] : A first Case of Meningitis/Encephalitis associated with SARS-Coronavirus-2. *International Journal of Infectious Diseases*.2020; 10.1016/j.ijid.2020.03.062 10.1016/j.ijid.2020.03.062 PMC719537832251791
